# Nest survival of Seaside Sparrows (*Ammospiza maritima*) in the wake of the *Deepwater Horizon* oil spill

**DOI:** 10.1371/journal.pone.0259022

**Published:** 2021-10-26

**Authors:** Megan E. Hart, Anna Perez-Umphrey, Philip C. Stouffer, Christine Bergeon Burns, Andrea Bonisoli-Alquati, Sabrina S. Taylor, Stefan Woltmann

**Affiliations:** 1 Center of Excellence for Field Biology, and Department of Biology, Austin Peay State University, Clarksville, TN, United States of America; 2 School of Renewable Natural Resources, Louisiana State University and AgCenter, Baton Rouge, LA, United States of America; The University of Southern Mississippi, UNITED STATES

## Abstract

In 2010, the *Deepwater Horizon* oil spill released an estimated 4.9 million barrels of oil into the Gulf of Mexico, damaging coastal ecosystems. Seaside Sparrows (*Ammospiza maritima*)—a year-round resident of Gulf Coast salt marshes—were exposed to oil, as shown by published isotopic and molecular analyses, but fitness consequences have not been clarified. We monitored nests around two bays in Plaquemines Parish, Louisiana, USA from 2012–2017 to assess possible impacts on the nesting biology of Seaside Sparrows. A majority of nests failed (76% of known-fate nests, *N* = 252 nests, 3521 exposure-days) during our study, and predation was the main cause of nest failure (~91% of failed nests). Logistic exposure analysis revealed that daily nest survival rate: (1) was greater at nests with denser vegetation at nest height, (2) was higher in the more sheltered bay we studied, (3) decreased over the course of the breeding season in each year, and (4) was not correlated with either sediment polycyclic aromatic hydrocarbon concentrations or estimated predator abundance during the years for which we had those data. Although the *Deepwater Horizon* spill impacted other aspects of Seaside Sparrow ecology, we found no definitive effect of initial oiling or oiled sediment on nest survival during 2012–2017. Because predation was the overwhelming cause of nest failure in our study, additional work on these communities is needed to fully understand demographic and ecological impacts of storms, oil spills, other pollutants, and sea-level rise on Seaside Sparrows and their predators.

## Introduction

Oil spills have detrimental and often long-lasting effects on wildlife populations [1–5]. On April 20, 2010, the *Deepwater Horizon* (DWH) oil platform exploded in the Gulf of Mexico, resulting in the uncontrolled release of an estimated 4.9 million barrels of oil over a period of 87 days. Ecosystems throughout the northern Gulf of Mexico were impacted [6–8], including ca. 1700 km of shoreline, 45% of which were wetlands [9, 10]. Of the coastal wetlands impacted by the DWH, 95% were in Louisiana, and the majority of these were salt marshes [10].

Oil exposure has detrimental direct and indirect effects on organisms, which range from death and acute, toxic effects to chronic or cascading impacts [4, 11–14]. The most immediate impacts of oil are from physical contact or ingestion [15]; an estimated 600,000–800,000 birds were killed during the DWH spill [16]. Negative impacts on bird populations also result from long-term exposure to polycyclic aromatic hydrocarbons (PAHs) via multiple pathways [17–19], or from changes in habitat quality or resource availability due to oiling [4, 13]. For example, following the Exxon *Valdez* spill in Alaska, Harlequin Duck (*Histrionicus histrionicus*) populations did not fully recover for at least 9 years post-spill [20], and experienced chronic exposure to oil for at least 20 years [17]. A combination of chronic exposure to PAHs and reduced food availability also depressed Pigeon Guillemot (*Cepphus columba*) populations for several years following the *Valdez* spill [2]. Given the greater challenge of cleaning oiled marshes compared to rocky shores of Alaska, it is reasonable to expect that PAHs will remain in marshes in various states of degradation along the Gulf of Mexico for many years [8, 21]

The DWH spill had both immediate and longer-term impacts on the salt marshes of Louisiana [8], and the severity of oiling varied along the Louisiana coast depending on currents and southern exposure of marsh edges to the open Gulf [10]. Edge vegetation of heavily oiled salt marsh died, leading to increased erosion and land loss in oiled areas [22], and PAH levels in marsh edge sediments remained elevated at least through 2018 [21]. Nearshore aboveground plant biomass and vegetation health both decreased on oiled plots [23], and some plant communities changed due to differential sensitivity to oiling [24]. Salt marsh animal communities including both invertebrates and vertebrates were impacted by the spill (e.g., arthropods [25], tabanid flies [26], snails [27], fishes [5, 28], birds [14, 29–32]). Nonetheless, some plant and animal communities appeared to recover within 4–7 years following the DWH spill [33–35].

The Seaside Sparrow (*Ammospiza maritima*) is endemic to salt marshes of the Atlantic and Gulf coasts of North America, and is abundant in southern Louisiana marshes impacted by the DWH spill [14, 36]. Seaside Sparrow populations in the region (*A*. *m*. *fisheri*; a non-migratory group) are considered good indicators of healthy salt marsh because although Seaside Sparrows are vulnerable to habitat loss, they may be relatively resilient to some other disturbances (e.g., hurricanes) because of their adaptations to a dynamic ecosystem [37, 38]. Effects of the DWH spill on Seaside Sparrows include mortality [39], activation of detoxification response [31], and changes in the expression of genes involved in liver regeneration and the regulation of energy homeostasis, among others [40]. Oiling of plant and arthropod communities may also have negatively impacted Seaside Sparrows via changes to nest site availability, and reduction or alteration of food resources (e.g., [8, 21–23, 25, 41–43]). In contrast, we expected that the DWH spill could have had a positive effect on Seaside Sparrow nest success via relief from predation pressure if the spill negatively impacted populations of marsh rice rats (*Oryzomys palustris*)—a main predator of Seaside Sparrow nests, and a species more consistently found in direct contact with the substrate. The DWH spill thus had the potential to affect Seaside Sparrow nesting success via a number of possible pathways, including direct, toxicological effects on adults, reduced availability of high quality nest sites, decreased prey availability, as well as possible relief from nest predation pressure from marsh rice rats. Within this landscape of marshes and bays, the degree of direct exposure to the Gulf of Mexico affects wave action, tidal inundation, and marsh plant and bird nest predator communities. In the wake of the DWH spill, direct southern exposure of marshes to the Gulf of Mexico also resulted in increased exposure to oil.

We monitored Seaside Sparrow nests in salt marshes of Louisiana from 2012–2017 to assess potential long-term impacts of the oil spill on Seaside Sparrow nest survival. We hypothesized that nest survival could have decreased as a result of oiling, and would increase over time as effects of the oil spill subsided. We asked whether Seaside Sparrow nest placement (i.e., microhabitat), sediment PAH concentrations, or rice rat abundance was associated with differences in nest survival. We also considered that direct southern exposure to the Gulf of Mexico (which exposes some sites to higher wind and wave energy, and in these circumstances to oil from the DWH spill) could influence nest survival via differences in plot vegetation, tidal inundation, and nest predator communities [44–46].

## Methods

### Ethics statement

Field work and animal handling were conducted in compliance with Institutional Animal Care and Use Committee (IACUC) permits at Louisiana State University AgCenter (sparrows: AE2011-04, A2012-05, A2015-04; marsh rice rats: A2013-09 and A2016-06) and Austin Peay State University (17–008). Birds were captured and banded under USFWS permit 22648 and Louisiana Department of Wildlife and Fisheries (LDWF) permits LNHP-17-039, LNHP-16-048, LNHP-15-033, LNHP-14-051, LNHP-13-059, LNHP-12-023. Marsh rice rats were trapped under Louisiana Department of Wildlife and Fisheries permit numbers LNHP-17-064, LNHP-16-056, LNHP-15-039, LNHP-14-052, and LNHP-13-060.

### Study area

**Fig 1:**
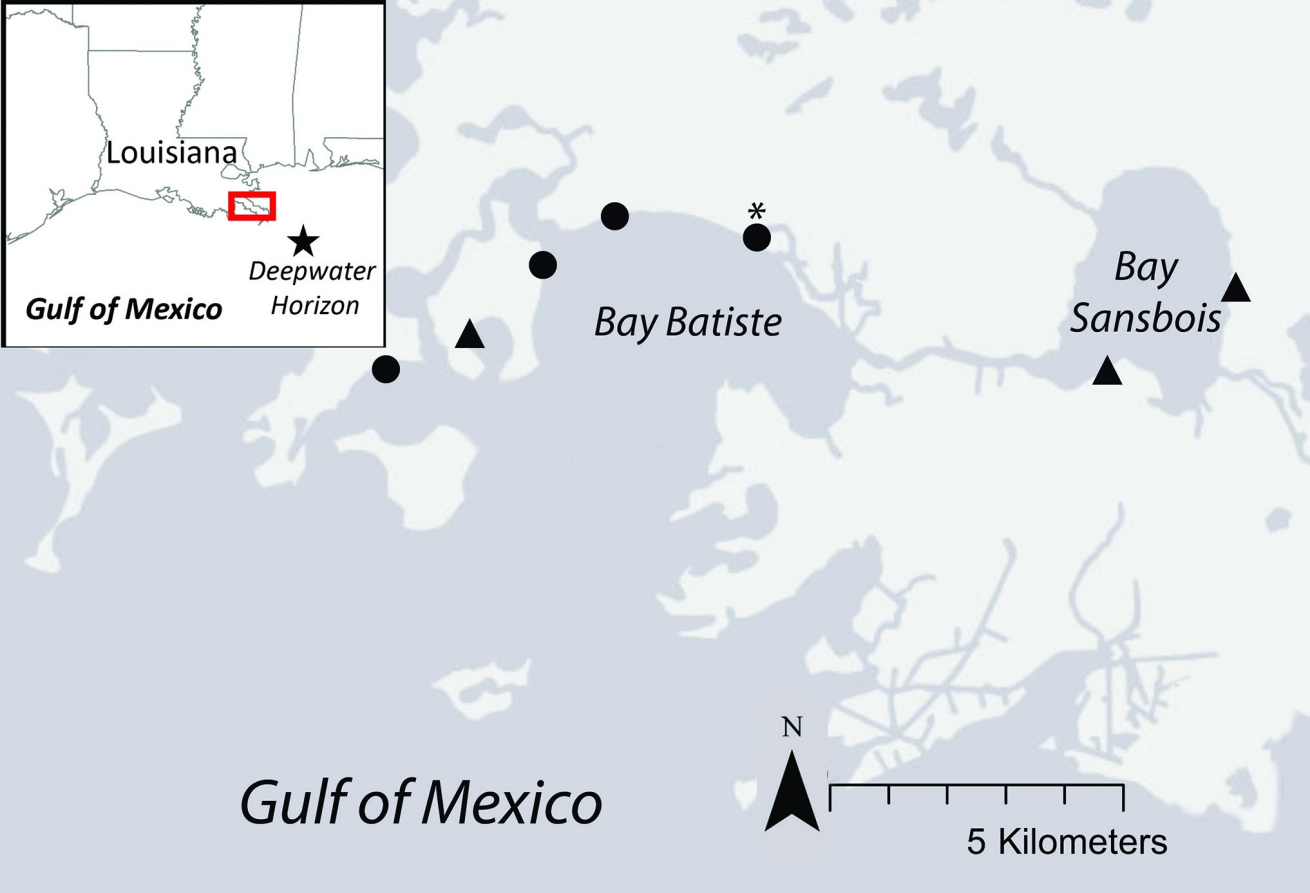
Location of Seaside Sparrow nest study plots (2012–2017) in the Barataria basin in Plaquemines Parish, Louisiana, USA. Circles represent sites with direct southern exposure to the Gulf of Mexico, and that were deemed initially oiled at the onset of our study. Triangles represent sites deemed initially unoiled. The site marked with an asterisk was added in 2013. The map was created with ArcMap^TM^ by ESRI.

Study sites were located in Plaquemines Parish, Louisiana (Fig 1). We selected study plots to encompass a range of initial oiling intensities with guidance from the Shoreline Cleanup and Assessment Techniques (SCAT) survey maps from 2010 in Bay Batiste and Bay Sansbois (https://erma.noaa.gov/gulfofmexico/erma.html#/, [10, 47]). Site context in the region could indirectly affect Seaside Sparrow nest success: Bay Batiste has considerable southern exposure to wind and wave action from the Gulf of Mexico, which influences the geomorphology and plant community of those sites. Bay Sansbois is not directly exposed to the Gulf of Mexico. In 2012, 3 plots were designated as unoiled (little to no oil present) and 3 plots were characterized as oiled (moderate to heavy oiling; [48]). A fourth heavily oiled plot was added in 2013 due to a scarcity of nests and adult Seaside Sparrows on initially oiled plots in 2012. Plot dimensions were 500 m along the marsh edge by 50 m perpendicular to the marsh edge; we selected only areas without canals, ponds or other extensively non-vegetated areas so that all plots had an equal area of potential nesting habitat. Salt marshes in the study area are dominated by three plant species: black needlerush (*Juncus roemerianus*), smooth cordgrass (*Sporobolus alterniflorus*), and saltgrass (*Distichlis spicata*).

### Nest searching and monitoring

From 2012–2017, we monitored nests annually from 15 March to 30 June, 2012–2017; this timeframe encompasses the majority of the Seaside Sparrow nesting season in this region. Search effort was equal (person-search-hours) across plots within years, but varied among years due to personnel constraints. Nests were located by systematic searching and using behavioral cues from adult Seaside Sparrows. Seaside Sparrows in our study have 12-day incubation and 9-day nestling periods, and a modal clutch size of 3 eggs (range 1–4; 132 of 252 nests had 3 eggs). Nests were monitored every 2–3 days until fate could be determined. Nests were classified as successful if they fledged at least 1 nestling as determined by: (1) sighting of fledglings near the nest; (2) adults carrying food to the area after the predicted fledge date; (3) begging sounds of fledglings in the surrounding vegetation; (4) the presence of fledgling fecal matter in or on the rim of the nest or on the surrounding vegetation [49], or (5) documentation of fledging via video monitoring (see below). Nests were classified as failed if: (1) eggs or nestlings disappeared from the nest before fledge day with no evidence of early fledging; (2) egg shells or nestling remains were present; or (3) nests were torn, tilted, flooded or otherwise destroyed before the fledge date. We used continuously recording camera systems on a subset of nests to identify nest predators in 2016 (*N* = 4) and 2017 (*N* = 16). Nests were randomly selected for video monitoring after first constraining the list of available nests to ones that were: (1) in incubation stage or with nestlings no older than 5 days (to avoid the risk of abandonment or early fledging), and additionally (2) in locations without temporary standing water (which made installing recording systems difficult). The monitoring system consisted of a small (ca. 12 x 6 cm) security camera with infrared LEDs for nighttime recording mounted on a garden stake (typically no more than 20 cm away due to dense vegetation), and a waterproof case containing a digital video recorder and batteries placed ≥ 25 m from the nest. Batteries and data storage cards were replaced every 5–7 days as long as the nest was active.

### Vegetation and sediment PAH measurements

Once nests were empty, we measured vegetation characteristics for each nest (Table 1) following a protocol developed by Lehmicke [50] for Seaside Sparrows. A pole marked in 20-cm increments was used to measure vegetation and nest height, and the number of stems (live and dead) touching the pole within 20-cm sections was recorded at the 4 corners of a 1m^2^ frame placed around the nest [51]. Percentage of ground cover was estimated for each plant species, and for open water, detritus, and bare ground within 1m^2^ and 5m-radius around the nest. For each nest, a random point was chosen 8–25 m from the nest to represent a potential nest site not chosen by the female but still within the male’s territory. The same vegetation survey was conducted for random points. To incorporate sediment PAHs in our models of nest survival we used the geometric mean of total aromatics measured from sediment samples collected on or near each plot in each year of our study (see Data Availability).

**Table 1:** Description of variables considered for Seaside Sparrow nest site selection and nest survival models in Louisiana, 2012–2017.

Variable	Description	Site selection	Survival
PAHs	Geometric mean (ng/g) of aromatics sampled from sediments collected on or near our study plots in each year (2013–2017 only)		x
Bay	Bay Sansbois (sheltered, not initially oiled) vs Bay Batiste (exposed, mostly initially oiled)	x	x
Stem0.20,	Number of vegetation stems sampled around a nest or random vegetation point; measured in 20-cm height categories	x	x
Stem20.40,		x	
Stem40.60,		x	x
Stem60.80,		x	
Stem80.100,		x	x
Stem100.120		x	
Day	Day of clutch completion (ordinal day of year; including Day^2^ and Day^3^)		x
Year	Treated as continuous (Year_con_), categorical (Year_cat_), or Year^2^ depending on model		x
*Juncus*	Amount (%) of vegetation type or cover (water, bare ground) in a 5m-radius plot around a nest or random point	x	x
*Sporobolus*		x	x
*Distichlis*		x	
Water		x	
BareGround		x	x
OtherCov		x	
Rats	Marsh rice rat abundance, derived from closed population mark-recapture models (2015–2017 only)		x

### Marsh rice rat trapping and abundance estimation

Marsh rice rats are a common small mammal in Gulf and Atlantic coast salt marshes, and they are the only muroid rodent in the marshes where we worked [52]. Marsh rice rats (‘rats’ hereafter) have been implicated as important predators of Seaside Sparrow nests [36, 53] and were trapped and marked as part of a separate research effort in our research consortium. Briefly, rats were trapped over three consecutive nights each year in April-May in 2015–2017 in trapping grids set along the marsh edge. Estimates of rat abundance used in subsequent analyses of sparrow nest survival were derived by model averaging top Huggins closed capture models in Program MARK v. 6.2 [54] for each year (see [55] for a full description of trapping and abundance estimation methods).

### Nest site vegetation analysis

Analyses of vegetation and nest survival (below) were conducted in R v.4.1.0 [56]. To characterize vegetation and nest site characteristics, we first identified and removed 1 variable from strongly correlated pairs (*r ≥* |0.5|), and we removed variables with near-zero variance (identified with *nearZeroVar* in the package *caret* [57]). Ratio scale data were scaled and centered prior to analyses. We used a logistic regression model to ask whether vegetation variables (see Table 1 for variable descriptions) varied between nest (coded as 1) and random points (coded as 0). We considered variables statistically significantly associated with nest placement if estimated 95% confidence intervals for their parameter estimates did not include 0.

### Analysis of nest survival

We used logistic exposure models [58] of nest survival in R package *glm2* [59] with a custom link function (http://rpubs.com/bbolker/logregexp) to analyze survival patterns of nests. We constructed models (Table 2) that can be broadly classified as addressing hypotheses related to: (1) nest concealment (factors immediately around a nest, such as vegetation); (2) landscape (factors such as bay characteristics and year-to-year variation); and (3) recovery (based on the expectation that nest survival could increase over time as potential effects of the DWH spill waned). The three classes of models are, of course, not mutually exclusive, but serve to identify important factors affecting nest survival without resorting to model dredging. To estimate exposure to oil we quantified the hydrocarbon content of the surface sediments (5-cm depth) by gas chromatography/mass spectrometry in selective ion monitoring mode (GC/MS-SIM), as previously described by Turner et al. [60]. We used sediment concentrations of PAHs as a proxy of exposure to oil, rather than a direct measure of blood concentrations of PAHs in birds because direct estimates would require large amounts of tissue and the sacrifice of individual birds, which is not compatible with the collection of life-history information. We included initiation day (ordinal Day of year, Day^2^, and Day^3^ of clutch completion, back-calculated from hatch date or nestling size when needed) in all models because it is commonly observed that DSR varies nonlinearly over the course of the breeding season (e.g., [61–63]). For nests that were found and failed during the incubation period, nest initiation date was set as the midpoint (day 6) of the incubation period [64]. Vegetation variables, rat abundance, and aromatic (PAH) measures were scaled and centered prior to analyses. We considered variables statistically significant if estimated 95% confidence intervals for parameter estimates did not include 0.

**Table 2:** Description and evaluation of six logistic exposure models explaining variance of Daily Survival Rates (DSR) of Seaside Sparrow nests in Louisiana, USA 2012–2017.

Model	Model Definition	Hypothesis	df	logLik	AICc	Δ AICc
F1	Bay + Year_cat_ + *Sporobolus* + *Juncus* + BareGround + Stems0.20 + Stems40.60 + Stems80.100	-	16	-174.1	382.50	0.00
R1	Year_con_ x Bay	Recovery	7	-186.8	388.06	5.56
L2	Year_cat_ + Bay	Landscape	10	-183.7	388.29	5.79
L1	Year_cat_ x Bay	Landscape	15	-179.3	390.58	8.09
C1	*Sporobolus* + *Juncus* + BareGround + Stems0.20 + Stems40.60 + Stems80.100	Concealment	10	-186.4	396.67	11.17
R2	Year^2^	Recovery	6	-194.6	401.57	19.08
Null	-	Constant DSR	1	-217.5	437.11	54.61

Models are broadly classified as testing Landscape, Recovery, or Concealment hypotheses. All models except the Null include Day (ordinal day of breeding season, including Day^2^ and Day^3^) as a covariate. See Table 1 for descriptions of variables.

Sediment PAH concentrations and rice rat abundances were only available for some years of our study (PAHs 2013–2017; rice rats 2015–2017), and we only used surveillance cameras on nests in 2016–2017. All three factors could influence nest survival. We tested for effects of sediment PAHs (geometric mean of aromatics), rice rat abundance, and camera presence on DSR by applying our best model of DSR from the 6-year dataset to the reduced datasets, and then compared that model to an identical model including PAHs, rice rats, or camera presence using likelihood ratio tests to ask if inclusion of the additional variables produced a better-fitting model.

## Results

### Vegetation characteristics and nest placement

**Fig 2:**
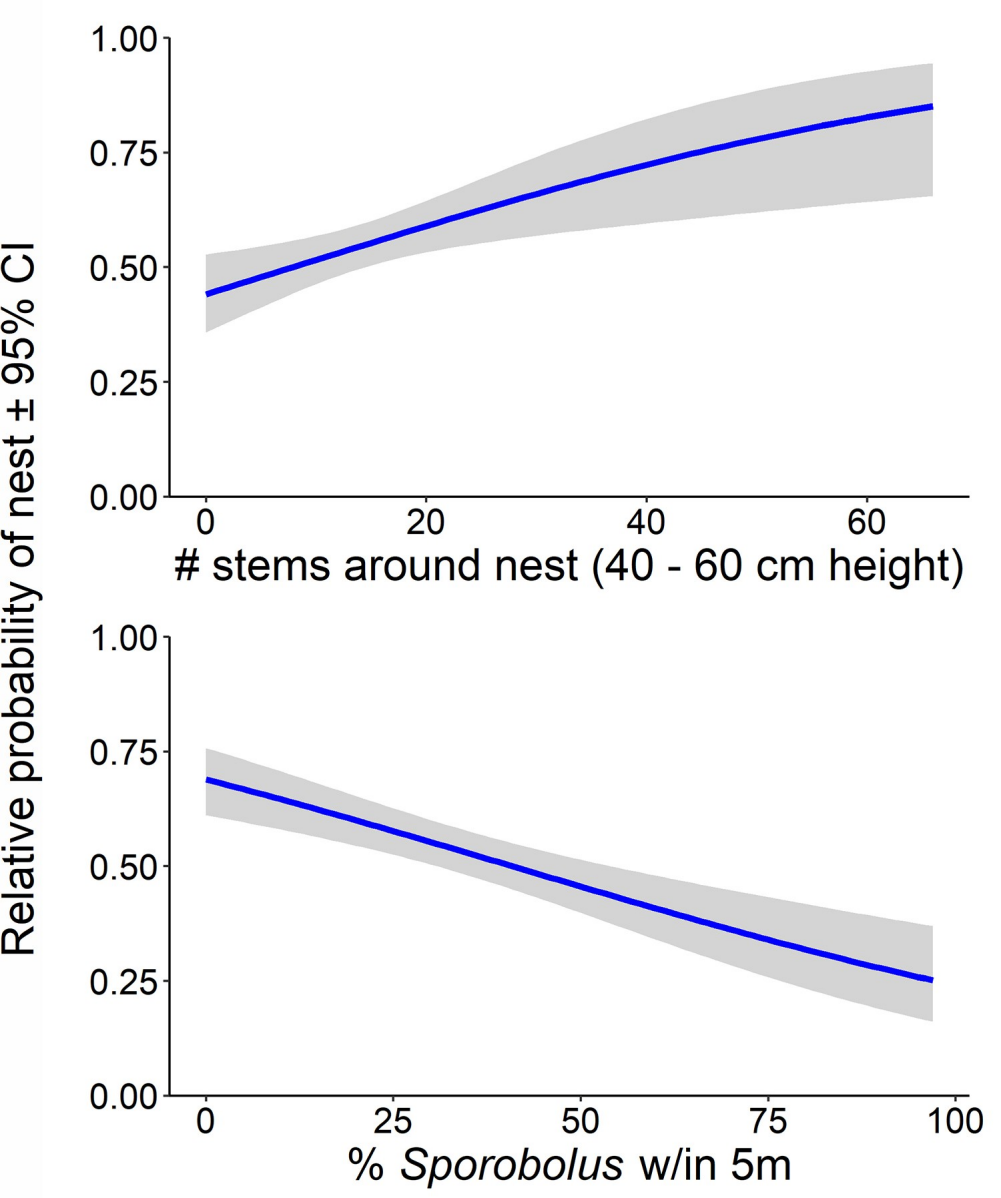
Relative probability (± 95% confidence intervals) of a vegetation sampling point being a Seaside Sparrow nest (vs a random point) considering two vegetation variables in Louisiana, 2012–2017. Relative to random locations within a territory, Seaside Sparrows were more likely to place their nests in sites with higher stem densities at mean nest height (~ 40 cm), and with less *Sporobolus* around the nest. Predicted estimates are derived from the full linear model, setting all other variables to their means, and Bay = “Batiste”.

When comparing nests to random locations, our model indicated that Seaside Sparrows disproportionately built their nests at points that had less *Sporobolus* cover, less bare ground cover, and a greater number of stems at nest height (40–60 cm) and near the ground (0–20 cm; Table 3, Fig 2). To ask whether initial oiling changed the vegetation community or structure on oiled plots, we ran the same model as above, but comparing nests within areas initially oiled or not in Bay Batiste (*N* = 194; there were no initially oiled locations in Bay Sansbois). Oiled nest points had less *Juncus* cover than unoiled points in one bay (Bay Batiste; β = -0.162, 95% CI -0.253, -0.071) but otherwise similar vegetation characteristics. Based on a model using only random points, the two bays differed slightly in their vegetation structure: Bay Batiste points had a greater number of stems at nest height (Stems 40–60 β = 0.069, 95% CI 0.004, 0.133), and fewer stems above nest height (Stems 80–100 β = -0.072, 95% CI -0.139, -0.006). Qualitatively, vegetation tended to be taller in Bay Sansbois, with higher stem densities in the overall shorter vegetation in Bay Batiste (S1 Fig).

**Table 3:** Parameter estimates from a model (logistic regression) of Seaside Sparrow nest site characteristics (vs random points) in Louisiana, USA, 2012–2017.

Variable	β	SE	95% confidence interval
Intercept	0.493	0.025	0.446, 0.541
*Juncus*	0.011	0.028	-0.065, 0.044
***Sporobolus***	**-0.137**	**0.028**	**-0.193, -0.082**
**BareGround**	**-0.048**	**0.023**	**-0.093, -0.003**
OtherCover	-0.023	0.023	-0.069, 0.023
Stems0.20	-0.056	0.028	-0.112, 0.000
**Stems40.60**	**0.075**	**0.025**	**0.027, 0.123**
Stems80.100	0.034	0.025	-0.014, 0.083
Bay (Sansbois)	0.030	0.055	-0.078, 0.137

Variables whose 95% confidence intervals did not overlap 0 are in **bold**. See Table 1 for variable descriptions.

### Nest survival

**Fig 3:**
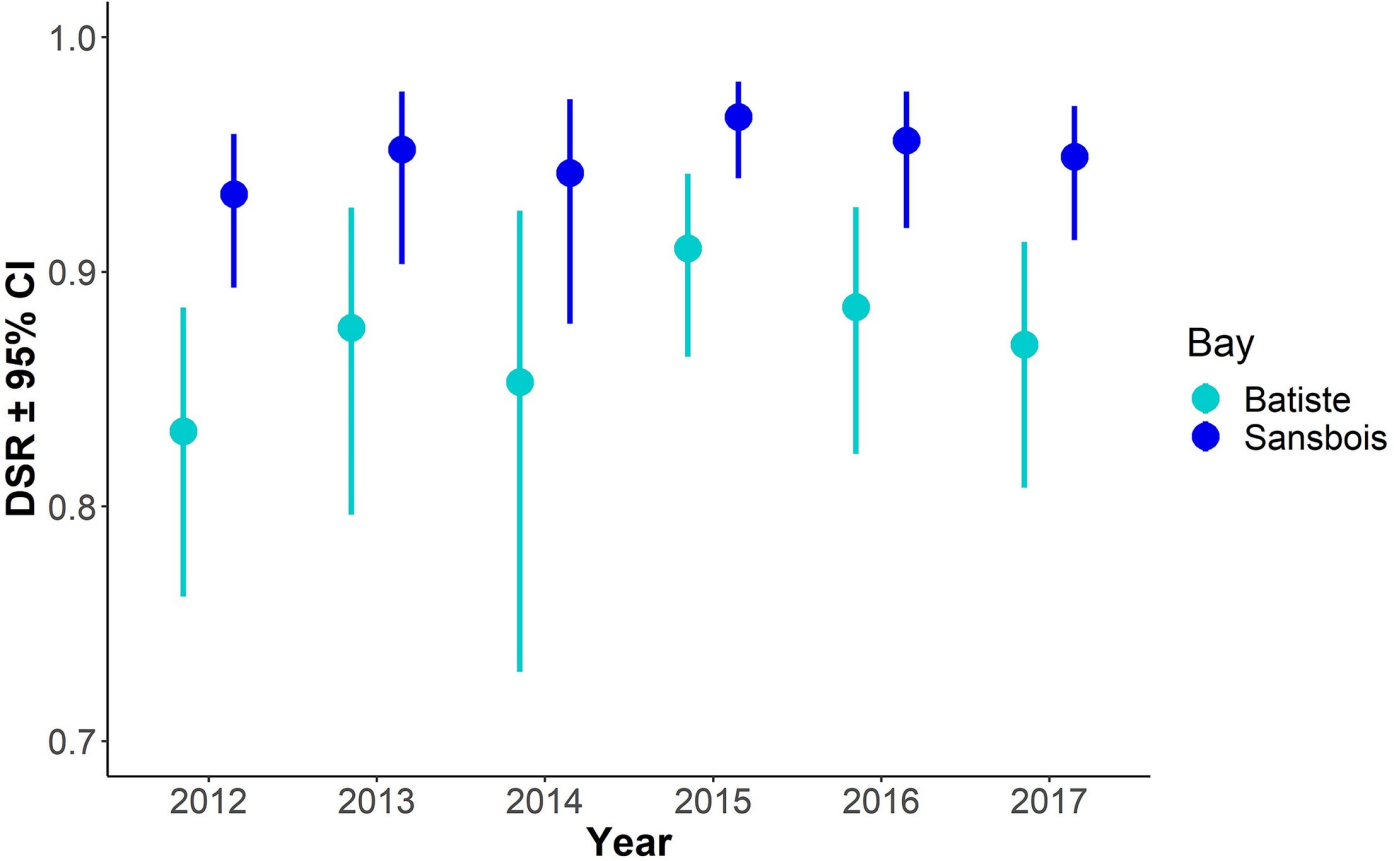
Mean estimated nest daily survival rates (DSR ± 95% confidence intervals) of Seaside Sparrows derived from a logistic exposure model in two bays during 2012–2017 in Louisiana, USA. DSR estimates are from a model including only Year and Bay.

**Fig 4:**
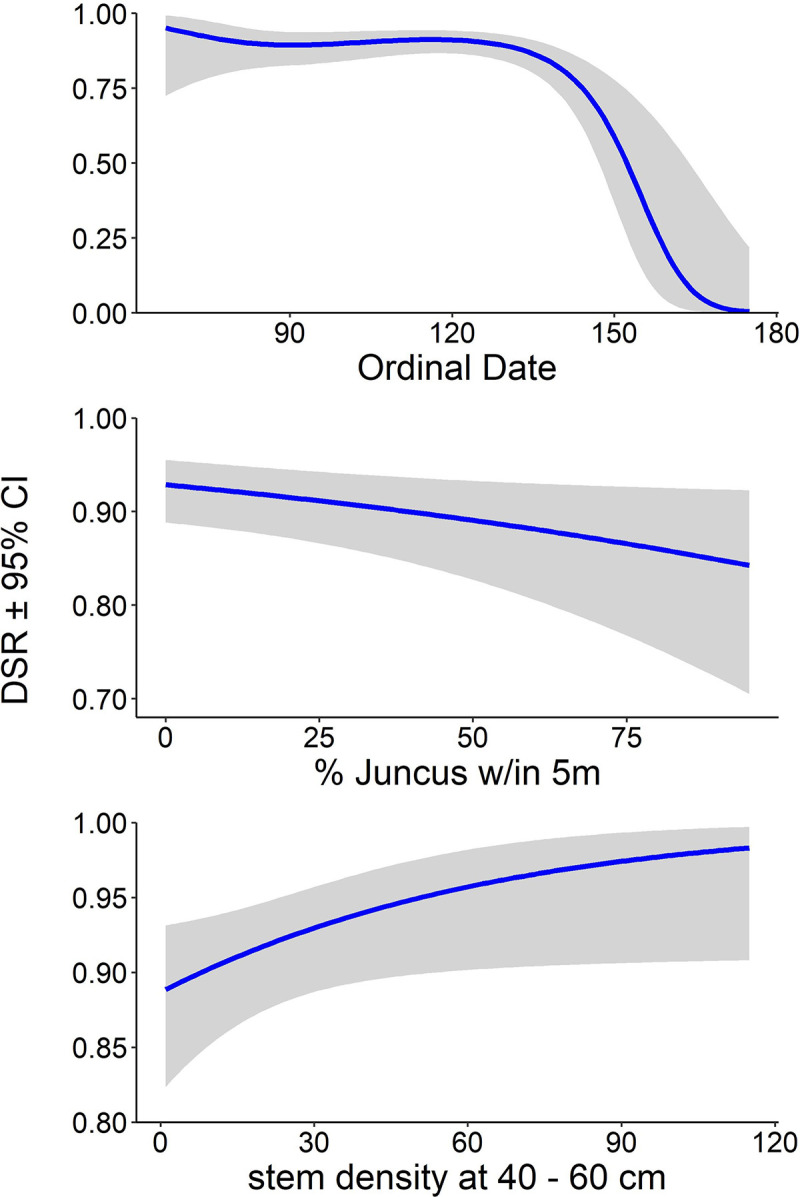
**Daily nest survival rates (± 95% confidence intervals) of Seaside Sparrows in Louisiana 2012–2017 as a function of Day (ordinal date) of nest initiation (top), % *Juncus* within 5 m of the nest (middle), and stem densities near the nest in the 40–60 height stratum (bottom).** Predicted estimates are derived from the top performing model of daily survival rates, using means for continuous variables, Year_cat_ = 2017, and Bay = Batiste.

Of the 252 nests in our study (encompassing 3521 exposure-days), 61 (24%) fledged at least one bird and 191 (76%) failed. Of the failed nests, 173 (91%) were depredated, 7 (4%) failed due to flooding, and 11 (6%) failed for uncertain reasons. Inclusion of sediment PAH (likelihood ratio test, *p* = 0.723), rice rat abundance (*p* = 0.910), and camera presence (*p* = 0.121) did not improve any of the models, so we present here only results from the full 6-year (2012–2017) dataset using models without PAHs, rats, or camera presence. Model selection indicated that the Full model accounted for the most variation in nest survival, outperforming the Recovery and Concealment models (Table 3). The Full model indicated that mean estimated daily nest survival rates: (a) were higher in Bay Sansbois in all years (Fig 3), (b) decreased over the course of the breeding season (Table 4, Fig 4), (c) decreased with increasing amounts of *Juncus* around the nest (Table 4, Fig 4), and (d) were greater at nests with a greater number of stems at nest height (Table 4, Fig 4).

**Table 4:** Parameter estimates from the best logistic exposure model of Seaside Sparrow nest survival in Louisiana, USA, 2012–2017.

Variable	β	SE	95% confidence interval
Intercept	1.60	0.223	1.15, 2.03
**Bay (Sansbois)**	**1.03**	**0.254**	**0.57, 1.52**
Year_cat_ 2013	0.359	0.374	-0.33, 1.08
Year_cat_ 2014	0.158	0.434	-0.64, 1.00
**Year**_**cat**_ **2015**	**0.717**	**0.312**	**0.14, 1.05**
Year_cat_ 2016	0.441	0.325	-0.15, 1.05
Year_cat_ 2017	0.292	0.304	-0.28, 0.88
**Day**	**-8.667**	**2.192**	**-12.94, -4.82**
**Day** ^**2**^	**-8.952**	**2.532**	**-13.72, -4.72**
**Day** ^**3**^	**-5.318**	**2.461**	**-10.38, -0.92**
*Sporobolus*	0.128	0.115	-0.09, 0.35
***Juncus***	**-0.259**	**0.123**	**-0.50, -0.03**
BareGround	0.154	0.109	-0.03, 0.35
Stems0.20	0.085	0.129	-0.16, 0.33
**Stems40.60**	**0.226**	**0.115**	**0.02, 0.45**
Stems80.100	-0.037	0.115	-0.27, 0.20

Parameter estimates whose 95% confidence intervals did not overlap 0 are in **bold**. See Table 1 for descriptions of variables.

### Nest predator identification

Of 20 nests monitored with video cameras, fledging was captured by video for 5 nests over both years. The other 15 nests failed due to predation (*N* = 9), flooding (*N* = 1), or unknown causes (failure without video documentation—animals sometimes moved the camera while approaching the nest: *N* = 5). We identified 3 nest predator species: rice rat (5 nests), American mink (*Neovison vison*; 4 nests), and squareback marsh crab (*Armases cinereum*, 1 nest). In the latter case, the crab foraged on one of the eggs (possibly causing abandonment by the sparrows), but the nest was subsequently also raided by a rice rat.

## Discussion

Despite numerous other responses to the *Deepwater Horizon* (DWH) oil spill in Seaside Sparrows and salt marsh ecosystems [8, 14, 29, 31, 32, 40, 65], our analyses revealed no definitive effects of marsh oiling on Seaside Sparrow daily nest survival rates in southeastern Louisiana in the 2 to 8 years following the DWH spill. Our daily nest survival estimates are low relative to other temperate passerines (e.g., [66–68]), but are within the range of other estimates for Seaside Sparrows [45, 46, 69]). Predation accounted for 91% of all known nest failures at our sites, in contrast to several studies on the Atlantic coast, where flooding is often the main cause of Seaside Sparrow nest failure [45, 46, 70] (but see also [71, 72]). We documented marsh rice rat and American mink as 2 main nest predators. Inclusion of rat abundance estimates did not improve models of nest survival, perhaps because other predators were also involved, or perhaps due to limited statistical power to detect an effect of rat abundance on nest survival. Although Seaside Sparrow nest survival was consistently lower on plots that had initially received oil from the DWH spill, inclusion of PAH measures did not improve our models of nest survival. Multiple factors lead us to conclude that differences due to site context—specifically southern exposure and thus the likelihood of receiving oil in the first place—are a key factor influencing predator and vegetation communities in these marshes. This may help explain why earlier coarse estimates of nest success suggested a decrease due to oiling [14]. Rather than being a direct effect of oiling, sites with southern exposure to the Gulf of Mexico differ from sites that do not by having slightly different vegetation structure and perhaps predator communities. Variation in exposure to PAHs and other pollutants among individual birds is also expected due to irregular distribution of contaminants, and to differences in habitat use and prey selection. Ultimately our results cannot exclude that inter-individual variation in exposure to oil and its toxicant constituents could account for some variation in nest survival, but if such an effect was present it was small relative to the effect of predation.

### Vegetation structure and nest placement

Our ability to explain variation in nest survival was improved by the inclusion of vegetation variables. Seaside Sparrow nests were associated with greater stem densities at typical nest height (mean nest height ~ 40 cm) and areas with less *Sporobolus* within 5m of the nest, suggesting selection for concealment; higher stem densities at typical nest height were associated with increased nest survival. We interpret nest placement in areas of high stem densities in our study as a mechanism to decrease predation risk (see, e.g., [73]). In contrast, greater vegetation density around nests has sometimes been associated with *increased* nest predation risk in other sparrow species [68, 74, 75], perhaps because the small mammals implicated as nest predators in those studies used dense vegetation as refugia, or because dense vegetation allowed small mammals easier climbing access to nests. Nest concealment from avian predators (i.e., those hunting from above) does not seem a likely mechanism for nest site selection in our study, as avian nest predators are largely absent from our study area during the nesting season.

Subtle differences in vegetation characteristics between bays included a near absence of *Distichlis*, and generally taller vegetation in Bay Sansbois (S1 Fig). These differences may influence predator communities, and are most likely pre-existing conditions not attributable to the DWH spill for several reasons: (a) oil effects on vegetation were most pronounced within 10 m of the marsh edge [76], and our plots extended to 50m from the edge; (b) lightly- and moderately-oiled vegetation had largely recovered by 2013 [77]; and (c) much of the most heavily-oiled marsh edge had eroded away by 2013 [22, 77, 78]. Within Bay Batiste, oiling did appear to decrease the abundance of *Juncus*, perhaps because this species is more sensitive to oiling [9], and had not fully recovered in heavily oiled areas up to 3.5 years after the oil spill [77].

### Temporal variation

Seaside Sparrow nest survival declined over the course of the nesting season. Reports of declines in nest survival over a season are common in temperate birds [63, 79–81], and Baiser et al. [61] noted that predation likelihood increased during the breeding season for Cape Sable Seaside Sparrows (*A*. *m*. *mirabilis*). An increase in rat and mink populations during the sparrow nesting season may explain increased predation rates, as both predator species have young entering the population at this time [82]. Young rice rats depend on a primarily carnivorous diet in order to grow [52, 83], and a seasonal shift in diet from vegetative to animal material over the spring and summer months has been documented in rice rat gut and fecal contents [83, 84].

In addition to consistently lower mean nest survival on southern-exposed plots, we observed considerable annual variation in mean DSR in the 2 bays over the 6 years of our study. Coastal marshes are highly dynamic systems, where unpredictable wind-driven tides, hurricane activity, precipitation, and changes in salinity could influence Seaside Sparrows both directly and indirectly (e.g., by impacting food or predator populations [8, 25, 43, 65]). In our study, average Seaside Sparrow nest survival was lowest on south-exposed plots in our first season (2 years after the oil spill), but a pattern of “recovery” was not evident over the course of the study; daily survival rates of nests were not different across years within each bay.

Consideration of exposure pathways is a key aspect to understanding effects of remaining oil residues from the DWH and other sources in these marshes [40]. The lack of an association between PAHs and Seaside Sparrow nest DSR is tempered by a number of important caveats. Hurricane Isaac, for example, passed over our study area in August 2012 and redistributed oil that had been sequestered in sediments [21, 29]. This complicated an original study design of “oiled” vs “unoiled” plots: the entire region became effectively “oiled,” albeit at lower concentrations compared to shortly after the spill [8, 21]. Perhaps equally important to study design considerations are the facts that (a) there are no marshes in our landscape that are devoid of natural- and anthropogenic-sourced sediment PAHs [21], and (b) the distribution of oil from the DWH was uneven and complicated by erosion over time (itself a consequence of killed vegetation) and poorly-documented clean-up operations, leading to patches of marsh with less oil than indicated by initial SCAT maps [22]. In retrospect, it should perhaps come as little surprise that documenting and understanding the distribution and ultimate fate of oil in a complex and dynamic system such as a salt marsh requires considerable effort and resources [21].

A dietary shift by Seaside Sparrows in 2013 to more aquatic- and benthic-derived food sources was probably the result of depleted above-ground invertebrates (e.g., spiders, lepidoptera, grasshoppers) following marsh submersion during Hurricane Isaac [65]. Seaside Sparrows throughout the study area in 2013 also showed increased exposure to PAHs, probably as a result of foraging on contaminated prey in contaminated sediments when above-ground prey in the vegetation was less abundant [31]. Perhaps unsurprisingly, these impacts did not appear to depress DSR in 2013, since predation remained the overwhelming cause of nest failures. If there were effects of lingering contamination from the oil spill on Seaside Sparrow nest survival during our study, they were small and difficult to detect relative to intense predation pressure on Seaside Sparrow nests.

Long-term data are needed to better assess the impacts of oil spills and other potential stressors on Seaside Sparrow reproduction. PAHs from DWH oil and other sources will remain in the study area for decades, and future resuspension and redistribution of these pollutants (including sequestered, largely undegraded oil) is all but certain [8, 60]. Initial challenges to our study included a lack of pre-spill data, and a lack of funding and logistical support to begin the study in 2010, when oil from the DWH first reached the marshes. Understanding the influences of inherent and often unrecognized small-scale variation in these salt marshes that dictated where oil accumulated (e.g., plot orientation (this study); currents and freshwater diversions [85]) remains important given the potential for future oil spills and other impacts on these coastal marshes [21, 86]. Rice rat and mink populations in the region remain understudied, but these species are key to understanding Seaside Sparrow population dynamics, as they are not only predators on bird nests, but are surely also affected by many of the same factors affecting Seaside Sparrow populations. We recommend increased efforts to study how hurricanes, oil spills, marsh geography, sea level rise, invertebrate and predator communities interact to influence Seaside Sparrow demography and populations over time.

## Supporting information

S1 Fig Vegetation height profiles (mean stem counts ±SE) at Seaside Sparrow nest and paired random points (N = 252 points each) associated with two bays in Louisiana, showing (a) vegetation structure differences between bays, and (b) evidence of nest site selection by Seaside Sparrows to favor denser vegetation at typical nest heights (20–80 cm).
(DOCX)
