# Supplementary material for: Nest survival of Seaside Sparrows (Ammospiza maritima) in the wake of the Deepwater Horizon oil spill
Source: PLoS One. 2021 Oct 26;16(10):e0259022. doi: 10.1371/journal.pone.0259022 (PMC8547620; doi:10.1371/journal.pone.0259022)
Supplement: S1 Fig — Vegetation height profiles (mean stem counts ±SE) at Seaside Sparrow nest and paired random points (N = 252 points each) associated with two bays in Louisiana, showing (a) vegetation structure differences between bays, and (b) evidence of nest site selection by Seaside Sparrows to favor denser vegetation at typical nest heights (20–80 cm). (DOCX) [file pone.0259022.s001.docx]

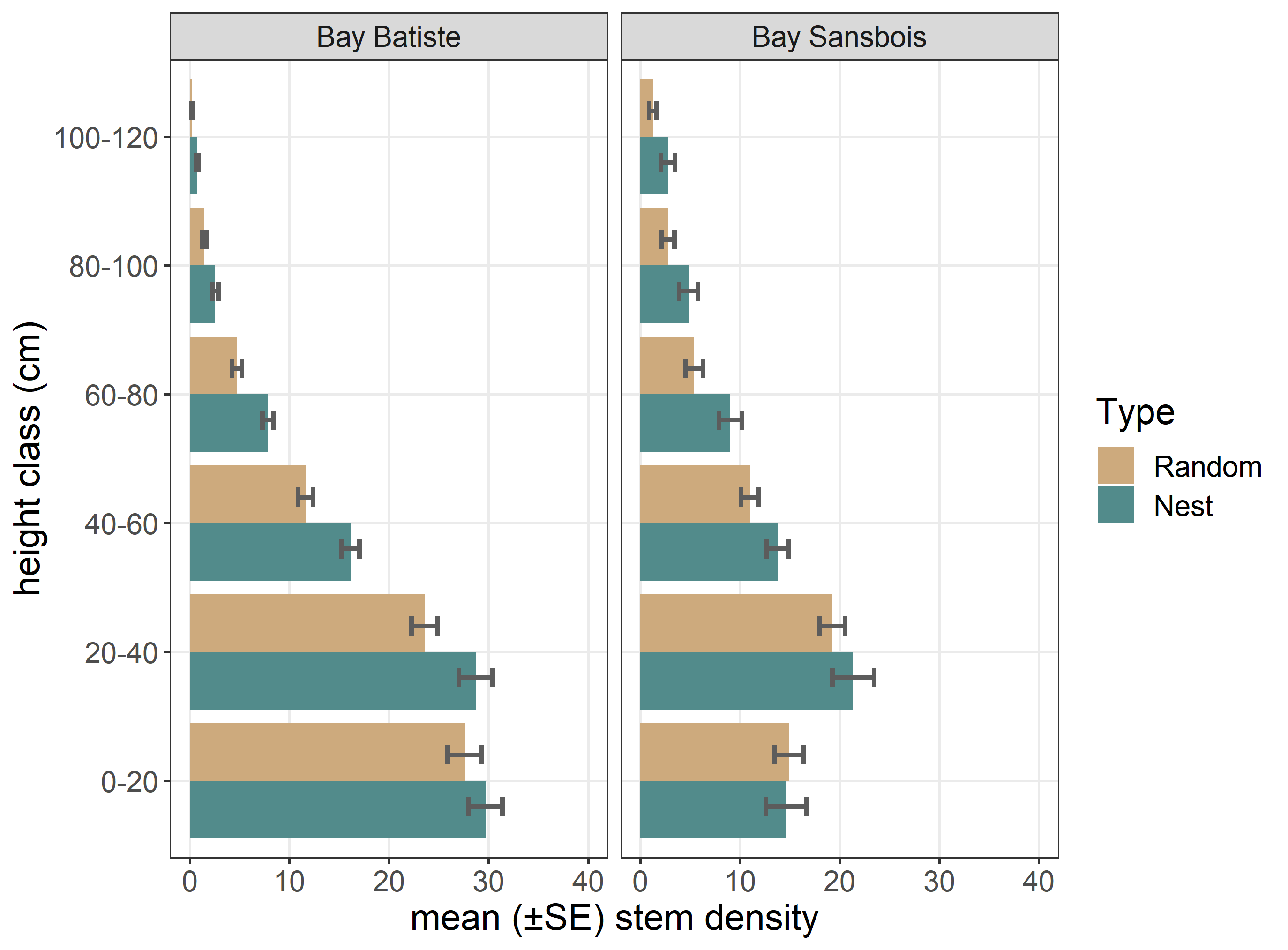


S1 Fig. Vegetation height profiles (mean stem counts ±SE) at Seaside Sparrow nest and paired random points (*N* = 252 points each) associated with two bays in Louisiana, showing (a) vegetation structure differences between bays, and (b) evidence of nest site selection by Seaside Sparrows to favor denser vegetation at typical nest heights (20-80 cm).
